# MOFormer: Self-Supervised Transformer Model for Metal–Organic Framework Property Prediction

**DOI:** 10.1021/jacs.2c11420

**Published:** 2023-01-27

**Authors:** Zhonglin Cao, Rishikesh Magar, Yuyang Wang, Amir Barati Farimani

**Affiliations:** † Department of Mechanical Engineering, Carnegie Mellon University, Pittsburgh, Pennsylvania15213, United States; ‡ Department of Chemical Engineering, Carnegie Mellon University, Pittsburgh, Pennsylvania15213, United States; ¶ Machine Learning Department, Carnegie Mellon University, Pittsburgh, Pennsylvania15213, United States

## Abstract

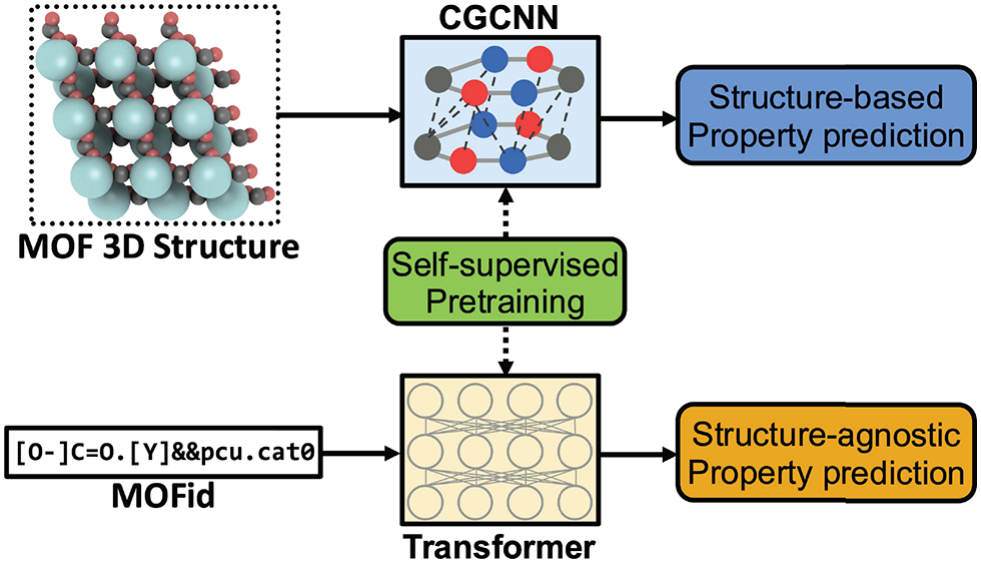

Metal–organic frameworks (MOFs) are materials with a high degree of porosity that can be used for many applications. However, the chemical space of MOFs is enormous due to the large variety of possible combinations of building blocks and topology. Discovering the optimal MOFs for specific applications requires an efficient and accurate search over countless potential candidates. Previous high-throughput screening methods using computational simulations like DFT can be time-consuming. Such methods also require the 3D atomic structures of MOFs, which adds one extra step when evaluating hypothetical MOFs. In this work, we propose a structure-agnostic deep learning method based on the Transformer model, named as MOFormer, for property predictions of MOFs. MOFormer takes a text string representation of MOF (MOFid) as input, thus circumventing the need of obtaining the 3D structure of a hypothetical MOF and accelerating the screening process. By comparing to other descriptors such as Stoichiometric-120 and revised autocorrelations, we demonstrate that MOFormer can achieve state-of-the-art structure-agnostic prediction accuracy on all benchmarks. Furthermore, we introduce a self-supervised learning framework that pretrains the MOFormer via maximizing the cross-correlation between its structure-agnostic representations and structure-based representations of the crystal graph convolutional neural network (CGCNN) on >400k publicly available MOF data. Benchmarks show that pretraining improves the prediction accuracy of both models on various downstream prediction tasks. Furthermore, we revealed that MOFormer can be more data-efficient on quantum-chemical property prediction than structure-based CGCNN when training data is limited. Overall, MOFormer provides a novel perspective on efficient MOF property prediction using deep learning.

## Introduction

Metal–organic frameworks (MOFs) are a type of porous crystalline materials,^1,2^ which have been extensively researched during the past several decades. Research interests have been induced by the porous structure and versatile nature of MOFs on their potential applications such as gas adsorption,^3−5^ water harvesting and desalination,^6−8^ and energy storage.^9−11^ MOFs typically consist of several building blocks, including metal nodes and organic linkers.^4,12,13^ The assembly of those building blocks following certain topologies generates the two-dimensional or three-dimensional porous structures of MOFs. Because of the countless possible combinations of metal nodes, organic linkers, and topologies,^13,14^ there is a sheer number of MOFs with different physicochemical properties and surface chemistries. Given the enormous variety of possible MOF structures, rapidly and inexpensively selecting the potential top performers for each specific task can be challenging. High-throughput screening with computational tools such as molecular simulation^5,15^ or density functional theory (DFT)^16,17^ has been widely used to evaluate the properties of MOFs. Without the need to experimentally synthesize MOF structures, those computational tools accelerate the screening process and allow researchers to screen hundreds of thousands of hypothetical MOF structures^4,5^ for their performance in different applications.

Recently, machine learning (ML) models have become increasingly popular in the field of MOF property prediction.^18−25^ The advantage of the ML models over the simulation methods is their instantaneous inference of the properties of MOFs. In contrast, the simulation methods require a computationally expensive rerun for every new MOF. In the past decade, multiple large-scale MOF data sets are released, including the CoRE MOF 2019,^26^ hypothetical MOFs,^5^ and QMOF.^27,28^ These data sets contain the atomic structures of MOFs and their computed properties like CO_2_ adsorption and band gap. These data sets are large enough to train accurate data-driven ML models for the prediction of MOF properties. Handcrafted geometrical features such as large cavity diameter and pore limiting diameter have been used as input to a multilayer perceptron (MLP) to predict MOF properties.^19,23^ Although the training of MLP with a few layers can be fast, such a method suffers from underwhelming accuracy due to the simplicity of network architecture. Moreover, selecting features requires extensive domain knowledge from the researchers and optimized 3D structures of MOFs, thus making this method less generic. Given the aforementioned drawbacks, a novel method that can achieve high accuracy with a more generic input of MOF representations should be pursued. Wang et al.^29^ utilize the crystal graph convolutional neural network (CGCNN)^30^ to predict methane adsorption of MOFs. CGCNN is a prevalent model which has an architecture designed specifically for crystalline materials. It takes the element type and the 3D coordinates of atoms in the crystalline materials as input and constructs a crystal graph. CGCNN can extract features that encode rich chemical information through convolution operations on the crystal graph. However, obtaining the 3D structures of MOFs is a necessity when using the structure-based CGCNN model. In addition, some large MOF structures consist of hundreds or even thousands of atoms, thus rendering crystal graphs of them memory-inefficient.

Enlightened by the fact that all MOFs are combinations of metal nodes, organic linkers, and topologies, Bucior et al.^31^ proposed a text string representation of MOFs called MOFid. The two core sections of a typical MOFid include the chemical information on building blocks and the topology and the catenation of the MOF structure. The building blocks are represented by an extensively used string representation of molecules called SMILES.^32^ The topology and catenation are each represented by a code adopted from the Reticular Chemistry Structure Resource (RCSR) database.^33^ Therefore, MOFid is a concise text string representation of MOFs that preserves the chemical and the majority of the structural information through topology encoding. The MOFid text-based representation enables the application of language ML models that take text string as input for MOF property prediction.

In this work, we proposed and developed a Transformer-based language model for MOF property prediction. Transformer and its variants have become the top choice for natural language processing tasks since the publication in 2017 by Vaswani et al.^34^ The multihead attention mechanism allows the Transformer model to learn contextual information in a sequence without suffering from long-range dependency.^35,36^ With its success in processing long sequential data, Transformer and its other variants are also adopted for chemistry or bioinformatics applications such as molecular^37−39^ and protein^40^ property prediction. The Transformer model in our work, named as MOFormer, takes a modified MOFid as input to make predictions of various MOF properties. The advantage of this method is that it does not require the 3D atomic structure of the MOF (structure-agnostic), thus enabling a much faster and more flexible exploration of the hypothetical MOF space. Specifically, MOFormer can be used to estimate properties of MOFs using only a hypothetically created MOFid. Predicting properties such as thermal conductivity is challenging for MOFormer because these properties are highly related to atom connections. However, we demonstrate that MOFormer can be the most accurate structure-agnostic model in predicting other properties such as bang gap and gas adsorption. In practice, pretraining the Transformer model in a self-supervised manner^40−44^ can leverage a large quantity of unlabeled data to help the model learn a more robust representation of the sequence and further improve its performance in downstream tasks. To take advantage of pretraining, we also added a self-supervised learning framework, in which the MOFormer and the CGCNN models are jointly pretrained with >400k MOF structures. Benchmarks show that pretraining improves the prediction accuracy of both models. Dimensionality reduction tools are used to visualize the latent representation learned by both models to provide insight into their performance characteristics. Visualization of attention weights in MOFormer demonstrates that MOFormer learns MOF representations based on some key atoms and topology. Lastly, we compared the data efficiency of models to show which one is a better choice when training data is limited.

## Methods

### MOFid Tokenization and Transformer

**Figure 1:**
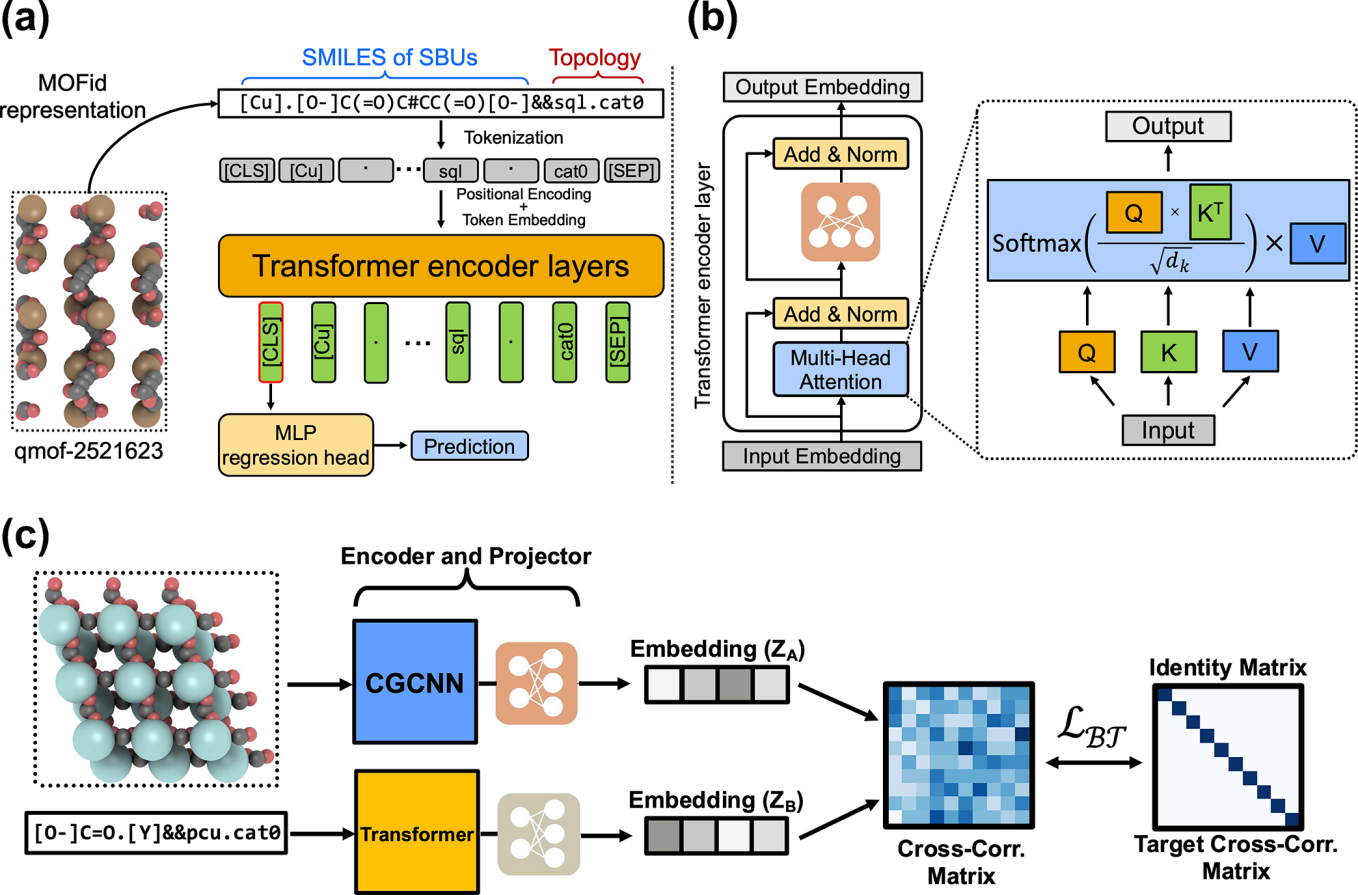
(a) The pipeline of the MOFormer model. A MOFid of a MOF (qmof-2521623 is used as an example) is the input to the model. The MOFid is converted into a tokenized sequence before being embedded and applied with positional encoding. The sequence is then fed into multiple Transformer encoder layers. The learned embedding of the first token will be used as input to an MLP regression head for downstream prediction tasks. (b) A schematic showing the details of each Transformer encoder layer. Embeddings of the sequence pass through the multihead scaled dot-product attention layer and then an MLP. Residue connection and layer normalization are adopted for both the attention and the MLP. (c) The self-supervised learning framework with CGCNN and MOFormer. The 3D structure and the MOFid of the same MOF are fed into the CGCNN and MOFormer, respectively, for representation learning. The MLP head following each models projects the representations into embeddings (*Z*_*A*_ and *Z*_*B*_). A cross-correlation matrix is then constructed using the embeddings. Barlow Twins loss is applied to optimize the cross-correlation matrix to be as close as possible to an identity matrix.

The MOFormer is built upon the encoder part of the Transformer model that takes a tokenized MOFid as input (Figure 1a). The MOFid tokenizer is a customized version of the SMILES tokenizer.^45^ The SMILES strings of all secondary building units (SBUs) of the MOFid are tokenized by the SMILES tokenizer, while the topology and catenation section of the MOFid is separately tokenized based on the topology encoding adopted from RCSR.^33^ The tokens from both sections are then connected by a separator token “&&”. The tokenization process follows the BERT^41^ to add a [CLS] token and a [SEP] token at the beginning and the end of the sequence to symbolize the start and the end, respectively. Since the tokenized sequences conform to a fixed length of 512, sequences longer than the fixed length are truncated, and sequences shorter than that are padded with special tokens [PAD]. None of the MOFids in the QMOF data set have a length over 512 tokens, and only 385 out of 102 858 MOFids (approximately 0.37%) in the hMOF data set have a length greater than 512 after tokenization.

A tokenized sequence is embedded and combined with a positional encoding (Figure 1a) to include information about the relative and absolute position of each token.^46^ The position encoding is calculated by
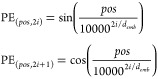
1where *pos* is the position of the token in the sequence, *i* is the index of dimension, and *d*_*emb*_ = 512 is the embedding dimension. The Transformer model is a deep neural network model built upon the self-attention mechanism (detailed in the Supporting Information). Each of the Transformer encoder layers consists of a multihead attention layer followed by a simple feed-forward multilayer perceptron (MLP). Residue connection^47^ and layer normalization^48^ are adopted for both the attention and the MLP. In each head of the attention layer (Figure 1b), the input sequence embedding *X* is multiplied with three learnable weight vectors *W*_*q*_, *W*_*k*_, and *W*_*v*_ to be converted to the query, key, and value vector (*Q*, *K*, *V*). The scaled dot-product attention *A* is then calculated by the equation:
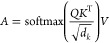
2where *d*_*k*_ is the dimension of *Q* and *K* (detailed in the Supporting Information). The randomly initialized *W*_*q*_, *W*_*k*_, and *W*_*v*_ vectors in each head allow the model to learn the contextual information between tokens in different representation subspaces.^34^ Attentions from all heads are concatenated together and then fed into the MLP for the projected output embedding, which has the same size as the input embedding. Given that the self-attention mechanism can incorporate the information on the whole sequence into each one of the token embeddings, theoretically, any one of the embeddings can be used as a representation of the whole sequence. Therefore, we followed the common practice of related works^37,38,49,50^ to use the embedding of the first token, [CLS], for further supervised learning tasks. The MOFormer model in this work contains six encoder layers. A smaller model with three layers has been benchmarked on the QMOF data set to show it has lower accuracy than the six-layer model (Table S4), thus leading us to select the six-layer model.

### Self-Supervised Pretraining with CGCNN

We introduce a self-supervised learning (SSL) paradigm for MOF representation learning. We designed the framework by taking into consideration two modalities of data including the text and graph information. One of the modalities is the text string representation (MOFid) that encapsulates building blocks’ stoichiometry and bonds (SMILES) and the topology of the MOF. The text string information is processed by the MOFormer. One of the limitations of text string data is the lack of information about the geometry and the neighborhood of atoms creating an information bottleneck for the text-string-input-based models. The structure-agnostic nature of the text string input can prevent the MOFormer from achieving higher performance than the graph-based models. To mitigate such a limitation of the MOFormer framework, we introduce SSL pretraining with CGCNN.^30^ The CGCNN model takes as input the 3D atomic structure of the MOF. The input to CGCNN contains the chemical information on all atoms in a MOF and the structure information in atomic resolution which is critical in property prediction tasks. To implement the SSL pipeline, we take inspiration from the Crystal Twins (CT) framework.^51^ The CT model makes use of the Barlow Twins loss function introduced by Zbontar et al.^52^ and SimSiam loss^53^ functions. In this work, we use the Barlow Twins loss function on the embeddings generated from the MOFormer and CGCNN encoder. As shown in Figure 1C, we initially encode both the text string representation and graph representation with their respective encoders. The MOFormer will encode the text string representation, and the CGCNN will encode the graph representation. We generate an embedding of size 512 from both the encoders and use it to generate the cross-correlation matrix following eq 3
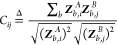
3where *b* is the batch index and *i*, *j* index the 512-dimensional output from the projector (*Z*^*A*^ and *Z*^*B*^), *A* is the graph representation, and *B* is text representation for the same MOF. Ideally, we want cross-correlation to be close to the identity matrix as both the representations generated from MOFormer and CGCNN are essentially capturing the same MOF. The Barlow Twins loss function, which we used for SSL pretraining (eq 4), tries to force the cross-correlation matrix to be the identity matrix.
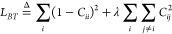
4where ***C*** is the cross-correlation matrix of embeddings from the MOFormer and CGCNN; the cross-correlation matrix is given by eq 3. The λ used in this work is set to 0.0051.

Finally, after pretraining the models using SSL, the encoder weights are shared during the finetuning stage (Figure S1). The pretraining hyperparameter details are shown in Table S3. For finetuning, we initialize the model with pretrained weights and train the model for 200 epochs to generate the final prediction (Hyperparameters: Tables S1 and S2). The MOFormer and CGCNN models are finetuned separately. We observed that using an SSL pretraining framework improves the results of both CGCNN and MOFormer consistently for all the data sets.

### Data Sets and Other Featurizations

Three public MOF data sets including the CORE MOF 2019,^26^ the hypothetical MOFs^5^ (hMOF), and the Boyd&Woo^4^ are combined to create a large data set for the SSL pretraining. The pretraining data set only includes MOFs with both 3D structure and MOFid available. Since we consider the MOFid as a unique descriptor of each MOF, identical MOFids are defined as duplicated and are removed from the pretraining data set. After the removal of duplicated MOFs, the final pretraining data set has 413 535 unique MOFs. In the downstream prediction task, the MOFormer and the CGCNN are trained on the quantum MOF^27,28^ (QMOF) and hMOF in a supervised manner. The QMOF data set contains 20 375 MOFs each with a label of a DFT-calculated band gap in eV. Only 7466 MOFs in the QMOF data set have a MOFid available. On the other hand, the hMOF has 137 652 MOFs, of which 102 858 have an available MOFid. The models are trained on hMOF with the labels of CO_2_ and CH_4_ adsorption in mol kg^–1^ at 0.05, 0.5, and 2.5 bar of pressure. The benchmark data sets are split into training, test, and validation sets with a ratio of 0.7–0.15–0.15. During the training, the model with the best validation performance is recorded and then tested with the test set. According to the splitting rule, MOFormer has 5226–1119–1119 QMOF data and 72000–15428–15428 hMOF data, while other models have 14262–3056–3056 QMOF data and 96356–20647–20647 hMOF data in the training, validation, and test sets, respectively. Although the MOFs with an available MOFid form a subset of both benchmark data sets, the subset with MOFid shares the same distribution and has approximately the same mean and standard deviation compared with the original whole data set (Figures S2 and S3 in the Supporting Information). Therefore, it is fair to compare the performance of MOFormer and other models.

We also benchmarked the MOFormer and CGCNN against other non-DL-based featurization methods such as the Smooth Overlap of Atomic Positions^54−56^ (SOAP) and the Stoichiometric-120^57^ features. SOAP is a structure-based featurization method, and the Stoichiometric-120 is a structure-agnostic featurization method based on the statistical properties of the MOF’s stoichiometric formula. The parameters used for creating SOAP features are included in the Table S5. It is worth mentioning that the SOAP matrix of each MOF is converted into a single feature vector using the inner average. In addition to SOAP and Stoichiometric-120, we also benchmarked the performance of the revised autocorrelations (RACs^13,58,59^) descriptor of MOFs. RACs are a descriptor based on the crystal graph and atom properties of MOFs. Since RACs do not require the 3D Cartesian coordinates of atoms as input, they can be considered as a structure-agnostic descriptor. RACs of MOFs are obtained using the mofdscribe(60) package. XGBoost^61^ model is used to make predictions using those handcrafted features.

## Results and Discussion

### QMOF

The first data set we benchmark models on is the QMOF data set, in which the label for each MOF is the DFT-calculated band gap. A lower band gap value results in better conductivity of the MOF. Accurate prediction of the band gap can help to identify conductive MOFs which are useful in energy storage applications.^62,63^ The accuracy of models follows the rank of CGCNN > MOFormer > SOAP > RACs > Stoichiometric-120 (Table 1). MOFormer has a 21.2 and 16.9% lower MAE compared with the Stoichiometric-120 and RACs, respectively. It is worth noting that structure-agnostic MOFormer outperforms structure-based SOAP with a smaller size of the training set, indicating that MOFormer is capable of extracting critical features from the MOFid for energy-related property prediction. The pretraining helps to reduce the mean absolute error (MAE) of CGCNN by 6.79% and MOFormer by 5.34%. The reduced error proves the improvement brought by the pretraining.

**Table 1:** Benchmark Performance of Different Models on the Band Gap Prediction of the QMOF Data Set^a^

	CGCNN_scratch_	CGCNN_pretrain_	SOAP	MOFormer_scratch_	MOFormer_pretrain_	Stoichiometric-120	RACs
MAE (eV)	0.275 ± 0.015	**0.256 ± 0.006**	0.424 ± 0.007	0.387 ± 0.001	**0.367 ± 0.005**	0.466 ± 0.011	0.441 ± 0.008

a Mean absolute error (MAE, in the unit of eV) and standard deviation of three runs of different initial seeds of each model are reported. The left three models are structure-based, and the right four models are structure-agnostic. The best performance of each category is marked as bold.

**Figure 2:**
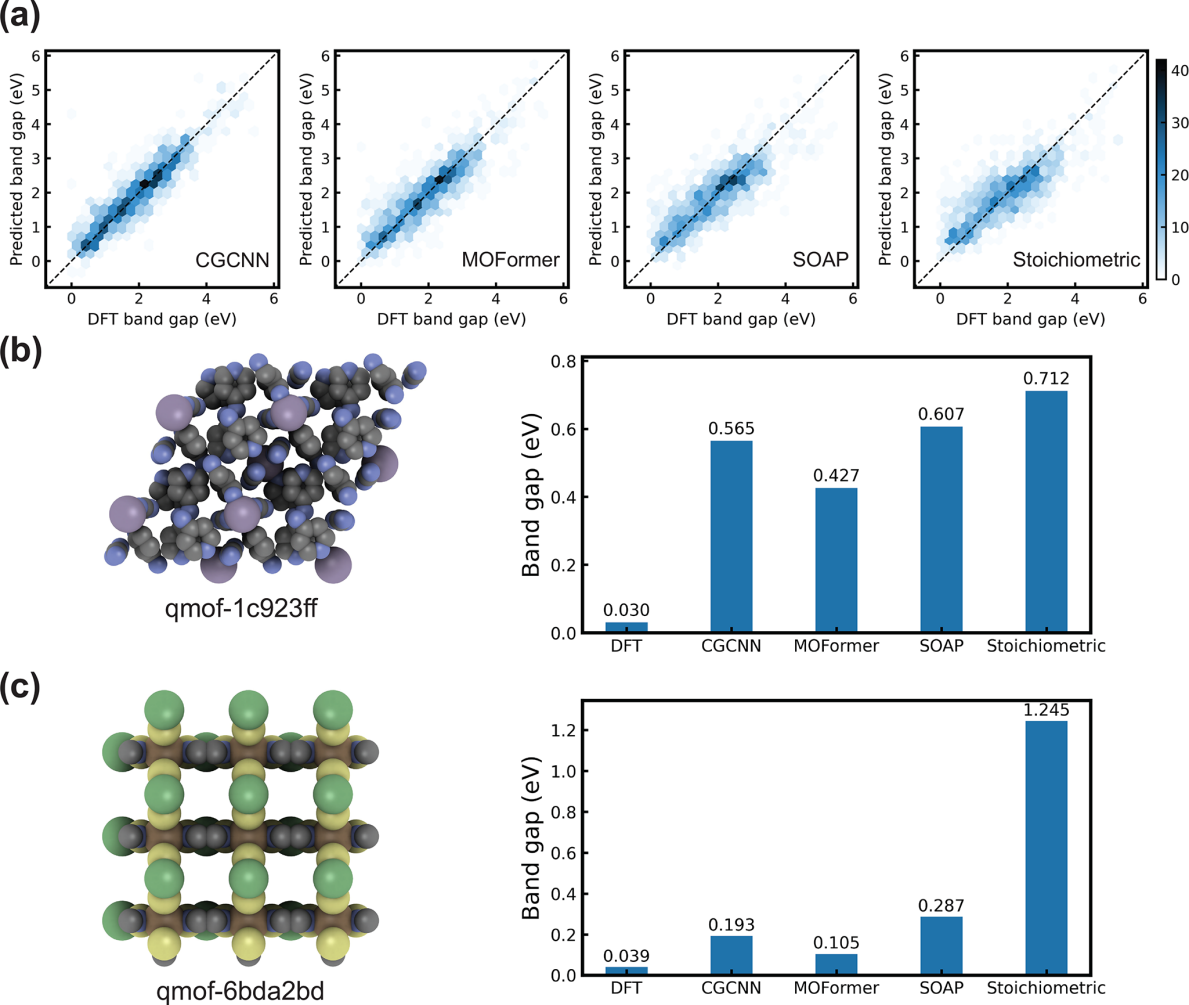
(a) The binned scatter plot shows the comparison between predicted and DFT-calculated band gap for MOFs in the QMOF data set. MOFs included in this figure are from the randomly split test set. The darker color of each hexagonal bin represents more data points in the bin. The dashed line represents perfect prediction. (b,c) Visualization^64^ of the MOF structure with lowest (qmof-1c923ff) and the second lowest (qmof-6bda2bd) band gap in the test set. The bar plot shows the comparison between predictions made by different models.

To better understand the superior performance of MOFormer and CGCNN in QMOF, we trained the four models with the same training set and then examined their performance on the same test set consisting of 1119 randomly selected MOFs. The binned scatter plot (Figure 2a) shows the comparison between the predicted and the DFT-calculated band gap. A darker color means more data fall in the bin. More predictions made by CGCNN and MOFormer are closer to the ground truth, especially for MOFs with a band gap ≤2 eV. The SOAP and Stoichiometric-120 are more likely to overpredict the lower band gap. This weakness of SOAP and Stoichiometric-120 can also be confirmed by the kernel density estimation of predicted values (Figure S4). The MOFs with the top-two lowest band gaps in this test set are the qmof-1c923ff (0.03 eV) and qmof-6bda2bd (0.039 eV). Band gaps predicted by MOFormer and CGCNN are much closer to the DFT-calculated value than predictions by SOAP and Stoichiometric-120 (Figure 2b,c), especially for qmof-6bda2bd. Accurately predicting the low band gap of MOFs can lead to the discovery of a conductive MOF, rendering pretrained MOFormer and CGCNN more valuable for prescreening MOFs.

### hMOF

The models are also benchmarked on the hMOF data set with the labels of CO_2_ and CH_4_ adsorption under 0.05, 0.5, and 2.5 bar of pressure. Table 2 shows that pretrained MOFormer is constantly outperforming Stoichiometric-120 by achieving a 35–48% lower MAE. Pretrained MOFormer also achieves a 25–42% lower MAE than RACs. Pretrained CGCNN outperforms other models for the CO_2_ adsorption prediction. The pretraining in average improves the accuracy of MOFormer by 4.3% and the CGCNN by 16.5% over all gas adsorption predictions. When obtaining the structure is relatively fast (e.g., using molecular mechanics optimization with UFF), CGCNN bears the promise for accurate gas adsorption prediction, which can be further improved by pretraining with MOFormer. It is worth mentioning that the prediction accuracy of MOFormer does not significantly drop with overlength MOFid (Figure S5a). SOAP has surprisingly low MAE for the gas adsorption prediction, outperforming pretrained CGCNN for two of the three CH_4_ adsorption predictions and the CGCNN trained from scratch for all gas adsorption predictions. The outstanding performance of SOAP on hMOF can be attributed to the low variation of elements included in the hMOF data set. Only 11 different elements are present among all 137 652 hMOFs, which is very limited compared to 79 in the QMOF data set. A smaller number of elements results in a much smaller and less sparse SOAP feature vector (SOAP feature has a size of 2772 for hMOFs and 19 908 for QMOFs with the same parameters), thus leading to the high prediction accuracy of the following XGBoost regressor. However, the high accuracy of SOAP can hardly be sustainable when it is used in exploring more diverse hypothetical MOFs. When more elements are included in the data set, the SOAP feature vector size and sparsity increase drastically, rendering the data and model too large to be accommodated by the memory of local machines and a drop in prediction accuracy (Table S6). MOFormer and CGCNN will not suffer from such an issue, since their inputs remain invariant with increasing types of elements in the data set, making them better choices when exploring more diverse chemical space for MOFs.

**Table 2:** Benchmark Performance of Different Models on Gas Adsorption Prediction of the hMOF Data Set^a^

	CO_2_0.05 bar	CO_2_0.5 bar	CO_2_2.5 bar	CH_4_0.05 bar	CH_4_0.5 bar	CH_4_2.5 bar
CGCNN_scratch_	0.126 ± 0.005	0.391 ± 0.017	0.818 ± 0.050	0.028 ± 0.001	0.121 ± 0.006	0.333 ± 0.017
CGCNN_pretrain_	**0.110 ± 0.001**	**0.330 ± 0.002**	**0.645 ± 0.003**	0.025 ± 0.001	**0.099 ± 0.001**	0.258 ± 0.008
SOAP	0.115 ± 0.002	0.339 ± 0.004	0.666 ± 0.003	**0.022 ± 0.001**	0.106 ± 0.001	**0.239 ± 0.002**
MOFormer_scratch_	0.178 ± 0.002	0.558 ± 0.001	1.000 ± 0.013	**0.034 ± 0.000**	0.174 ± 0.002	**0.385 ± 0.003**
MOFormer_pretrain_	**0.158 ± 0.001**	**0.545 ± 0.008**	**0.982 ± 0.011**	**0.033 ± 0.000**	**0.161 ± 0.011**	**0.384 ± 0.003**
Stoichiometric-120	0.282 ± 0.002	0.983 ± 0.005	1.895 ± 0.003	0.050 ± 0.001	0.269 ± 0.001	0.631 ± 0.002
RACs	0.248 ± 0.002	0.842 ± 0.004	1.681 ± 0.004	0.044 ± 0.001	0.236 ± 0.002	0.570 ± 0.004

a Mean absolute error (mol kg^–1^) and standard deviation of three runs of different initial seeds of each model are reported. The top three models are structure-based, and the bottom four models are structure-agnostic. The best performance of each category is marked as bold.

**Figure 3:**
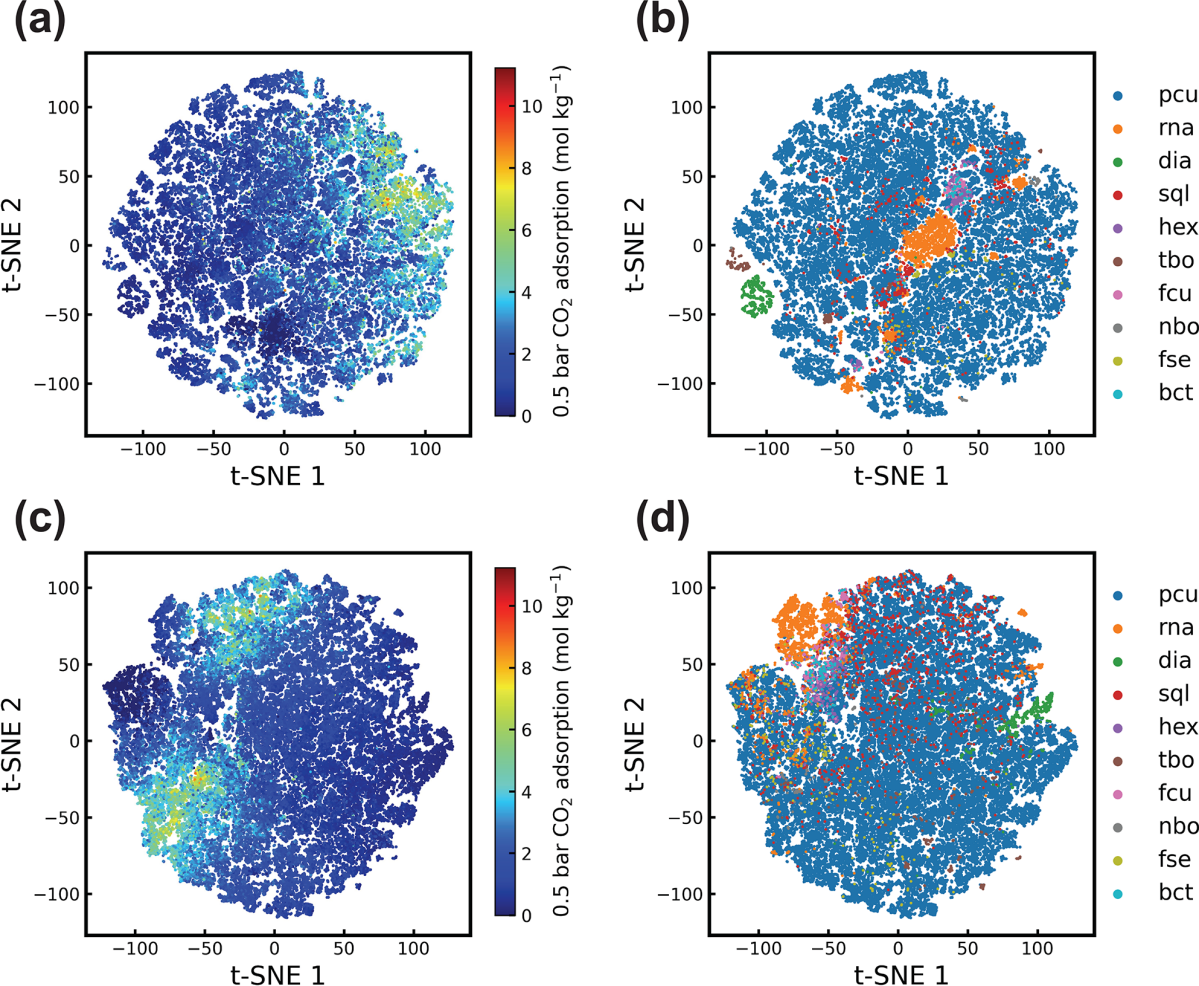
T-SNE^65^ dimension-reduced visualization of MOF representations learned by (a,b) the MOFormer and (c,d) the CGCNN. Each data point in (a) and (c) is colored by its CO_2_ adsorption at 0.5 bar of pressure, and each data point in (b) and (d) is colored by its topology. Only the MOF which has the top-10 most common topologies in the hMOF data set is shown.

The representations of MOFs learned by the MOFormer and CGCNN after finetuning are visualized to provide interpretability to the models (Figure 3). Each representation is projected to the 2D space using the dimension reduction tool t-SNE.^65^ t-SNE clusters more similar data points together while placing less similar data points further away. Only MOFs which have the top-10 most common topologies in hMOF are included in Figure 3 because they take >99.7% of the whole data set. We can observe that CGCNN representations cluster MOFs with high CO_2_ adsorption more closely than MOFormer representations by comparing Figure 3a and 3c. This contributes to the higher prediction accuracy of CGCNN. On the other hand, MOFormer representation clusters MOFs with the same topology closer than CGCNN representation does. For example, the MOFs with dia (green) and tbo (brown) topologies form two clusters in the lower left corner of the MOFormer representation visualization (Figure 3b). Those MOFs are much more loosely clustered in the CGCNN representation visualization (Figure 3d). The MOFormer representation focusing on topology can be caused by the fact that gas adsorption is more dependent on the 3D structure of the MOF compared with its atom composition. The only structure-related information contained in the MOFid is the topology encoding. Therefore, more weights are on the topology after MOFormer is finetuned to predict the gas adsorption of MOFs. The input of CGCNN is the 3D structure of MOFs with atomic resolution; thus, CGCNN can rely less on the topology for gas adsorption prediction. The MOFormer model representations may fail to accurately predict the properties of MOFs with rare topologies (Figure S5b). Such a disadvantage can be alleviated by in the future increasing the topology diversity in the training data set.

### Visualization of Attention Weights

**Figure 4:**
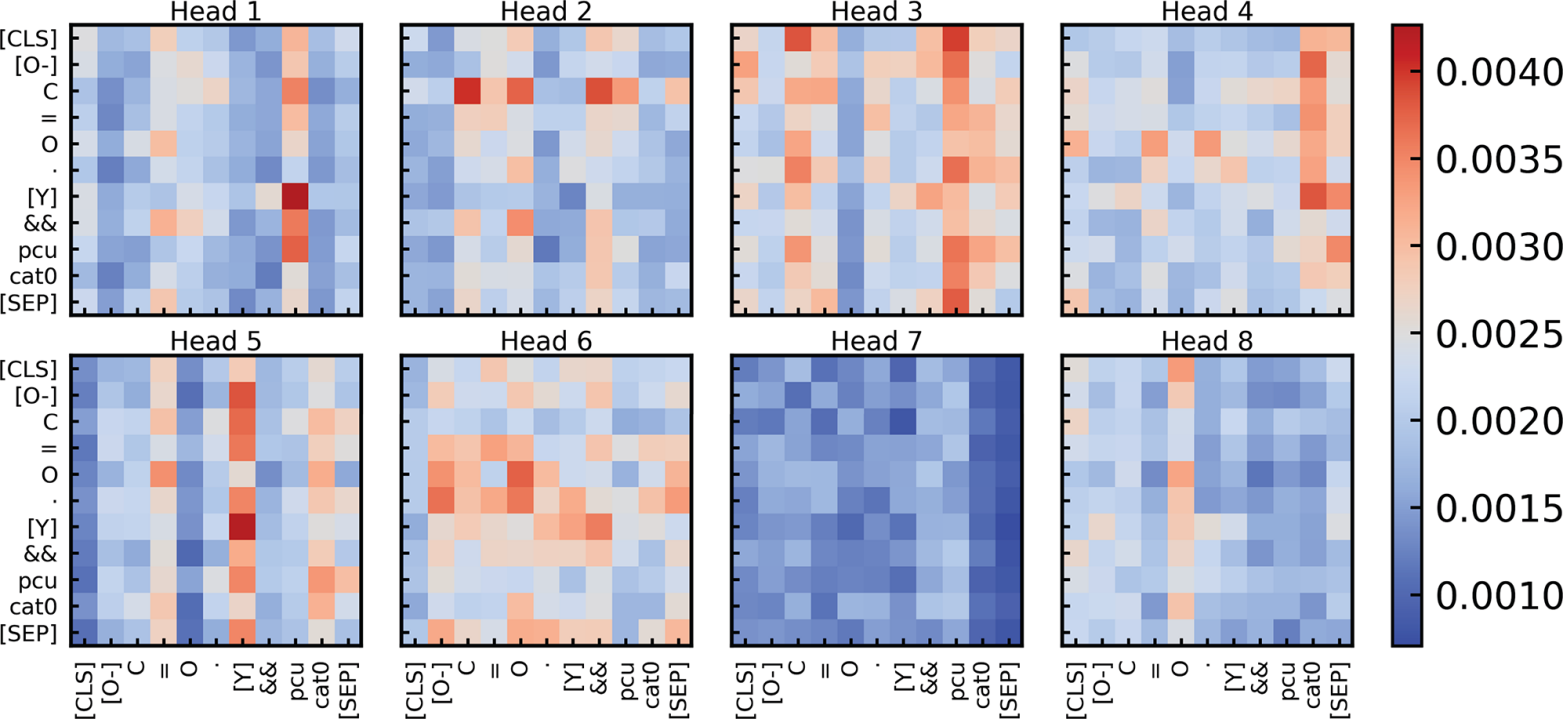
Heatmap of the attention between tokens (MOFid of qmof-ba40858) in different heads of the last MOFormer layer. We index each block in the heatmap as block_*n*,*i*,*j*_, where *i*, *j*, and *n* are the row, column, and head index, respectively. The value of block_*n*,*i*,*j*_ represents the attention on the *j*-th token from the *i*-th token in *n*-th heatmap.

Figure 4 demonstrates the attention maps between tokens of a MOFid (qmof-ba40858) in the last MOFormer layer after finetuning on band gap prediction. The attention map can serve as a visual interpretation of how the MOFormer learns MOF representations.^66^ We observe a strong attention in head 5 from all tokens to the metal node ytterium, and to the topology encoding pcu in heads 1 and 3. The attention from the metal node to the topology encoding is especially high in head 1. Moreover, a large attention weight can be observed between tokens in the SMILES of the SBUs in head 6. Heads 1, 3, 5, and 8 also show large attention weights on the carbon and the oxygen atom and the double bond in the organic building block. The attention weight visualization shows that MOFormer learns a representation that emphasizes the contextual information between key components in the MOFid including important atoms (e.g., Y, O, and C) and the topology, thus leading to more accurate prediction.

### Data Efficiency Comparison

**Figure 5:**
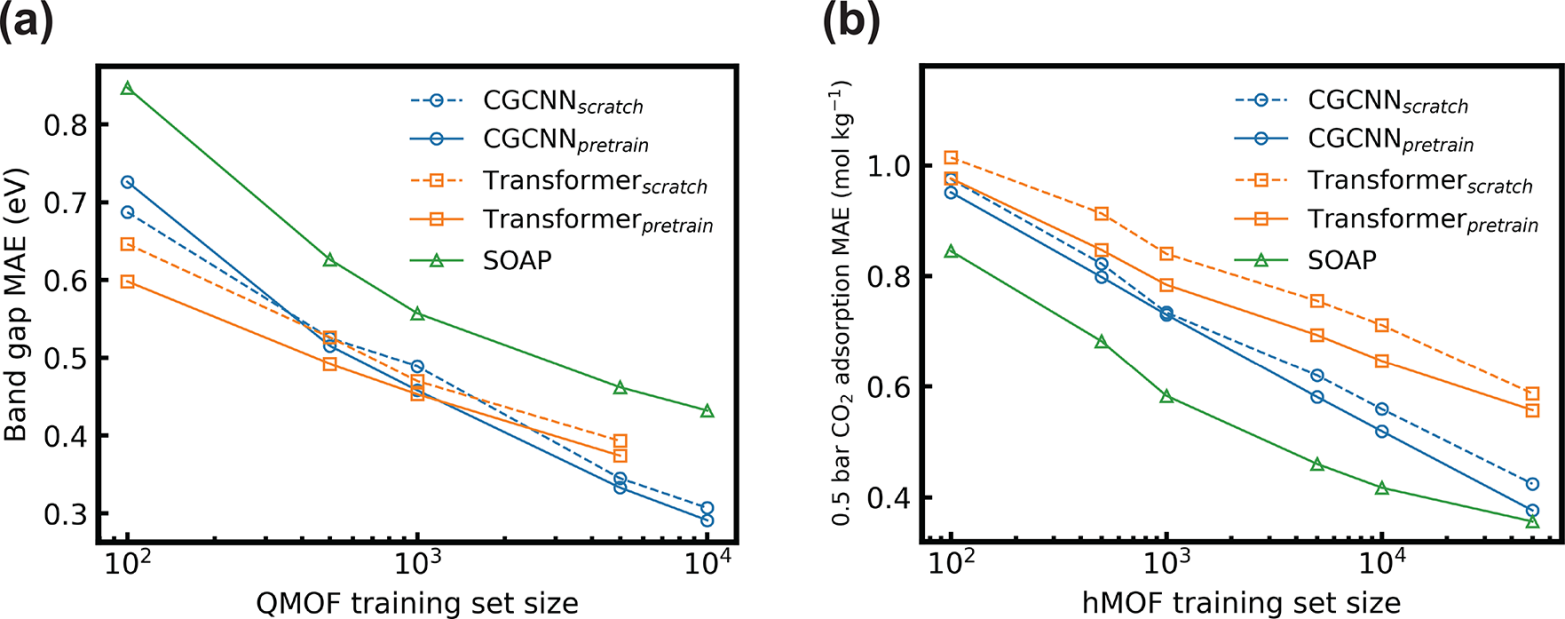
Data efficiency comparison between different models on the (a) QMOF and the (b) hMOF data set. The models are trained on a subset of the training set, while the validation and test set are kept the same. The subset sizes are 100, 500, 1000, 5000, 10 000, and 50 000 (hMOF only). Since only less than 7500 MOFs in QMOF have an available MOFid, the maximum training subset size for MOFormer on QMOF is 5000. Each data point is averaged over the results of three runs on randomly selected subsets drawn with different initial seeds.

Obtaining high-quality MOF data using experimental or DFT methods can be time-consuming and expensive. A model with high data efficiency is ideal when the training data size is limited. We compared the data efficiency of different models on the QMOF and hMOF data sets (CO_2_ adsorption at 0.5 bar pressure). For band gap prediction (Figure 5a), the pretrained MOFormer outperforms CGCNN when the training set size ≤1000. This makes MOFormer more valuable in predicting quantum-chemical properties when the training data set is difficult to build (i.e., experimentally synthesized MOFs). Both MOFormer and CGCNN achieve higher accuracy than SOAP regardless of the training set size on the QMOF data set. For CO_2_ adsorption prediction (Figure 5b), CGCNN constantly achieves higher accuracy than MOFormer regardless of the training set size, indicating its higher data efficiency. CGCNN outperforms MOFormer on hMOF because the CO_2_ adsorption correlates more with the MOF structure and the input to CGCNN provides more structural information than MOFid. SOAP achieves higher data efficiency than CGCNN and MOFormer on hMOF but is eventually caught up by CGCNN after the training set size exceeds 50 000. Figure 5a,b shows that pretraining consistently improves the data efficiency of MOFormer and CGCNN. Moreover, SOAP is shown to have diminishing improvement with increasing training set size, but CGCNN and MOFormer do not suffer from such an issue.

## Conclusion

In summary, we propose a Transformer-based model, named as MOFormer, for structure-agnostic MOF property prediction. Taking only MOFid as input, the MOFormer model is expected to expedite the exploration of hypothetical MOFs. We also introduce a self-supervised learning framework to jointly pretrain the MOFormer and CGCNN model on a large unlabeled MOF data set to enhance their prediction accuracy in downstream tasks. Compared with other structure-agnostic methods Stoichiometric-120 and RACs, MOFormer achieves 21.4% and 16.9% higher accuracy on band gap prediction as well as 35–48% and 25–42% higher accuracy on various gas adsorption prediction tasks, respectively. MOFormer even outperforms the structure-based SOAP method in band gap prediction with less training data. The pretraining is further shown to improve the accuracy of MOFormer by 5.34% and 4.3% on average and CGCNN by 6.79% and 16.5% on average, for band gap and gas adsorption prediction, respectively. MOFormer and CGCNN are shown to be less likely to overpredict the band gap of MOFs compared with SOAP and Stoichiometric-120, making them better choices for prescreening conductive MOFs for energy applications. When used for gas adsorption prediction of MOFs, MOFormer relies more on the topology information compared with CGCNN because of the strong correlation between the label and the structure of MOF. Visualization of the attention weights in the last MOFormer layer reveals that the attention layers in MOFormer focus more on several important atoms and the topology to learn the representation of a MOF. Lastly, MOFormer is shown to be more data-efficient than CGCNN for band gap prediction when the training set size ≤1000. As a structure-agnostic model, MOFormer can make rapid and accurate inferences on the property of MOFs (especially for quantum-chemical properties) using an arbitrarily constructed MOFid as input. Therefore, MOFormer can serve as a tool for exploring the vast chemical space of hypothetical MOFs.

## Data Availability

The Python code as well as data used in this work can be found on GitHub: https://github.com/zcao0420/MOFormer.

## Supporting Information Available

Transformer and self-attention mechanism. Details of CGCNN and MOFormer model. Details of the self-supervised pretraining. Effect of model size on prediction accuracy. Distribution of the benchmark data sets. Parameters for SOAP feature vector creation and the effect of SOAP vector length on model accuracy. Kernel density estimation of band gap prediction from different models. Prediction accuracy with extra long MOFid or rare topology (PDF)
