# Supplementary material for: MOFormer: Self-Supervised Transformer Model for Metal–Organic Framework Property Prediction
Source: J Am Chem Soc. 2023 Jan 27;145(5):2958–67. doi: 10.1021/jacs.2c11420 (PMC10041520; doi:10.1021/jacs.2c11420)
Supplement: Supplementary file 1 — ja2c11420_si_001.pdf [file ja2c11420_si_001.pdf]

MOFormer: Self-Supervised Transformer model for Metal-Organic Framework Property Prediction

Supporting Information

Zhonglin Cao,[†,§] Rishikesh Magar,[†,§] Yuyang Wang,[†] and Amir Barati Farimani[*,†,‡,¶]

†*Department of Mechanical Engineering, Carnegie Mellon University, Pittsburgh PA, USA 15213*

‡*Department of Biomedical Engineering, Carnegie Mellon University, Pittsburgh PA, USA 15213*

¶*Machine Learning Department, Carnegie Mellon University, Pittsburgh PA, USA 15213*

§*Joint First Authorship*

E-mail: barati@cmu.edu

Contents

1 Self-attention and the Transformer model **S3**

2 Training Details **S5**

2.1 MOFormer S5

2.2 CGCNN S5

2.3 SSL - Pretraining S6

2.4 Effect of MOFormer model size S7

3 Distribution of the benchmark datasets **S8**

4 Parameters to create SOAP feature vector **S10**

5 Comparison between models' performance in QMOF dataset **S11**

6 Prediction accuracy with extra long MOFid or rare topology **S12**

References **S14**

1 Self-attention and the Transformer model

In this work, we use the encoder part of the Transformer model as the base for the MOFormer framework. Transformer[1] model was proposed as a language model for sequence-to-sequence language translation. The encoder part of it learns a latent representation of the input sequence using the self-attention mechanism. Self-attention mechanism is previous used along with sequential models (such as recurrent neural network[2] and LSTM[3]) to combat the drop in model performance with long sequence. Transformer model drop the recurrent architecture in previous sequential models, and rely solely on the self-attention mechanism to learn the representation of the input sequence. The advantage of such model architecture is threefold. First, self-attention has lower per-layer computational complexity than recurrent architectures. Second, models can be highly parallelizable with only self-attention (as self-attention can be calculated using matrix multiplication) thus can be trained much faster. Third, long-range dependencies in the input sequence can be easily learned using self-attention mechanism. In MOFormer, the tokenized input sequence is embedded using the `nn.Embedding` function from the Pytorch[4] library with the vocabulary size of 4021 and embedding size of 512.

In the self-attention mechanism, each token in the sequence is linearly projected into its corresponding query, key, and value vector (Q, K, and V matrix for the entire sequence, respectively). The weights that linearly project the entire input sequence are denoted as the W_q, W_k, and W_v matrices. In this work, followed by Vaswani et al.,[1] the scaled dot-product attention:

$$Attention(Q, K, V) = \text{softmax}(\frac{QK^\top}{\sqrt{d_k}})V \tag{1}$$

is adopted. Multihead attention is to have multiple linear projection matrices in parallel for the calculation of attention. The W_q, W_k, and W_v in each attention head is initialized differently, thus multihead attention enables the model to learn representation of the input sequence from different subspaces. Assuming we have h attention heads, the multihead

attention is calculated as:

$$\begin{aligned} MultiheadAttn(Q, K, V) &= \text{Concat}(head_1 \dots head_h)W_o \\ \text{where } head_i &= Attention(Q_i, K_i, V_i) \end{aligned} \tag{2}$$

W_o is a matrix to linearly project the concatenated attention from each head; Q_i, K_i, and V_i is the query, key, and value matrix of the i-th head, respectively. In this work, $d_{emb} = 512$ and the number of head $h = 8$. The dimension of the W_q^i, W_k^i, and W_v^i (linear projection matrices in the i-th head) is $W_q^i \in \mathbb{R}^{d_{emb} \times d_k}$, $W_k^i \in \mathbb{R}^{d_{emb} \times d_k}$, $W_v^i \in \mathbb{R}^{d_{emb} \times d_v}$, where $d_{emb} = 512$ and $d_v = d_k = d_{emb}/h = 64$. Moreover, $W_o \in \mathbb{R}^{d_{emb} \times d_{emb}}$. The Transformer encoder in this work contains 6 self-attention layers (Figure 1b in the manuscript), in which the MLP has a dimension of 512.

2 Training Details

In this section, we describe in details the hyperparameters that we used for training the models. Our framework consists of MOFormer (transformer), CGGNN and the SSL pretraining done to improve the performance of both the GNN and the transformer model. During the finetuning stage, a MLP regression head is attached to the encoder part (MOFormer or CGCNN) to make prediction. The regression head has 4 layers, with 512, 256, 128 and 64 neurons in each layer, respectively.

2.1 MOFormer

The $\text{MOFormer}_{\text{Finetuning}}$ hyperparameters are shown in Table S1. Different learning rate (LR) are used for the encoder part (Transformer encoder) and the MLP regression head. We also train the $\text{MOFormer}_{\text{Scratch}}$ to evaluate the improvement in performance after SSL pre-training.

Table S1: Finetuning hyperparameters for the MOFormer model. LR indicate the learing rate

Hyperparameters	Batch Size	$\text{LR}_{Encoder}$	LR_{MLP}	Optimizer	Epochs	Embed. Size	Train/Val	Weight Decay
$\text{MOFormer}_{\text{Scratch}}$	64	0.00005	0.01	Adam	200	512	0.7/0.15/0.15	10^{-6}
$\text{MOFormer}_{\text{Finetuning}}$	64	0.00005	0.01	Adam	200	512	0.7/0.15/0.15	10^{-6}

2.2 CGCNN

The CGCNN model is structure based model that takes the ".cif" file as input. The MOFormer is a structure agnostic method, however, information like neighbourhood of atoms and geometry of the crystal are often lost in the text representation. To ensure we also have a structure based benchmark to predict MOF properties we also evaluate the performance of the CGCNN model.[5] The CGCNN model with its information rich features often outperforms the structure agnostic MOFormer. We also pretrain the MOFormer and the CGCNN together using the self-supervised learning. The performance of pretrained $\text{CGCNN}_{\text{Pretrain}}$

model is found to be higher than $CGCNN_{Scratch}$ model trained from Scratch without pre-training. The hyperparamters we used for the model are shown in Table S2.

Table S2: Finetuning hyperparameters for the CGCNN model. LR denotes the learning rate for the models.

Hyperparameters	Batch Size	Number of layers	LR	Optimizer	Epochs	Embed. Size	Train/Val/Test	Weight Decay
$CGCNN_{Scratch}$	128	3	0.01	Adam	200	512	0.7/0.15/0.15	10^{-6}
$CGCNN_{Finetuning}$	128	3	0.002	Adam	200	512	0.7/0.15/0.15	10^{-6}

2.3 SSL - Pretraining

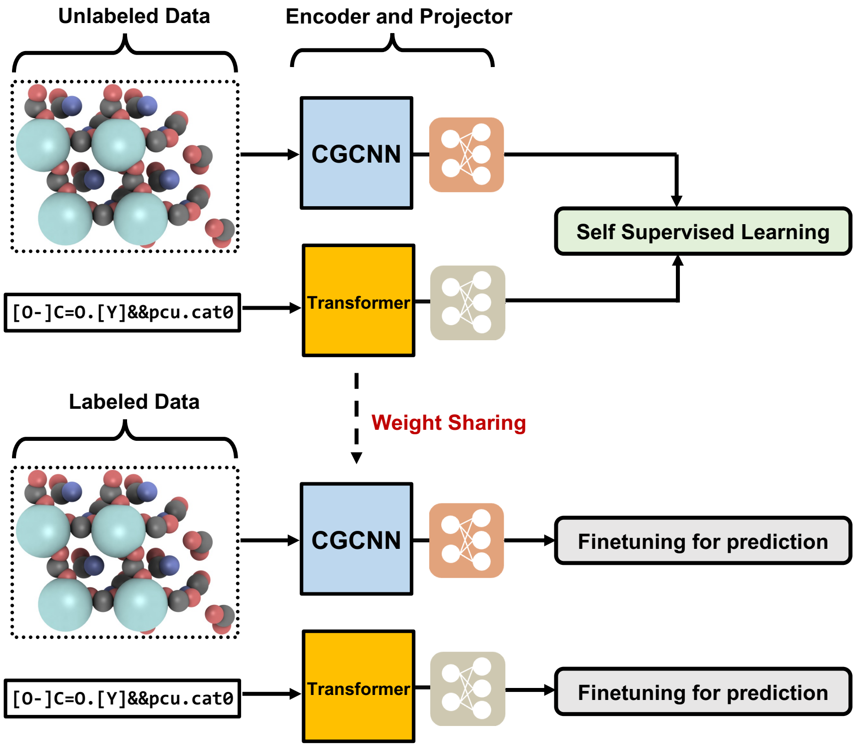

Figure S1: The SSL pretraining and finetuning framework. In this work, we use self supervised learning to pre-train both the CGCNN model and the MOFormer. The pre-trained weights are then shared during finetuning for down stream tasks. Using such a technique help us improve the perfromance of both the CGCNN and MOFormer.

Structure agnostic models often lack the geometry and atom neighborhood information leading to an information bottleneck for these models. To mitigate this issue, we develop the SSL pre-training framework. In the SSL pre-training framework we have the MOFormer

branch and the CGCNN branch, the goal of the SSL pre-training is to ensure that the representations from both the MOFormer and the CGCNN are similar. The pre-training helps both the MOFormer model and CGCNN in a way that it tries to leverage the embeddings learnt by the other model. The model benefit mutually from each other and the performance enhancements can be seen for both models during the finetuning stage (Figure S1). The hyperparamters we used during pretraining the model are shown in Table S3. The hyperparameters for the pretraining with SSL are inspired from Crystal Twins framework.[6]

Table S3: Hyperparameters for Pretraining the model.

Hyperparameters	Batch Size	Learning Rate	Optimizer	Epochs	Embed. Size	Train/Val
$\text{CGCNN}_{\text{Pretrain}}$	32	0.00001	Adam	15	512	0.95/0.05
$\text{MOFormer}_{\text{Pretrain}}$	32	0.00001	Adam	15	512	0.95/0.05

2.4 Effect of MOFormer model size

We tested the effect of MOFormer model size by training a smaller model which has only 3 Transformer encoder layers ($\text{MOFomer}_{\text{small}}$) on the QMOF dataset. The averaged MAE over 3 training of $\text{MOFomer}_{\text{small}}$ is 0.391 with standard deviation as 0.009. Compared with the regular MOFormer (QMOF MAE 0.387±0.001), $\text{MOFomer}_{\text{small}}$ has lower accuracy and less stable (higher standard deviation) result across multiple runs of training. Therefore, having a regular Transformer architecture with 8 heads and 6 layers can be justified.

Table S4: QMOF band gap prediction accuracy of MOFormer with 2 different sizes.

Model	Num. heads	Num. layers	QMOF MAE (eV)
$\text{MOFomer}_{\text{small}}$	8	3	0.391±0.009
$\text{MOFomer}_{\text{regular}}$	8	6	0.387±0.001

3 Distribution of the benchmark datasets

Since only part of MOF in each QMOF[7] and hMOF[8] dataset have available MOFid, we plot the distribution of the whole datasets and the subsets with MOFid. Figure S2 shows that the difference between the average band gap value between the whole dataset and the subset is as small as 0.135. The distribution between the data are also similar.

Figure S3 shows that the difference between the average CO_2/CH_4 adsorption value between the whole dataset and the subset is very small. The distribution between the data are also similar. Therefore the benchmark results comparisons between MOFormer and other models on both datasets are fair.

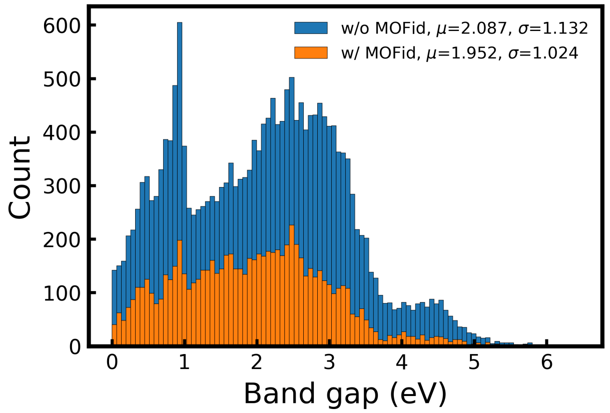

Figure S2: Histogram showing the distribution of QMOF dataset. Blue bars are all MOFs in the QMOF dataset. Orange bars are MOFs with MOFid available. Mean μ and standard deviation σ are reported.

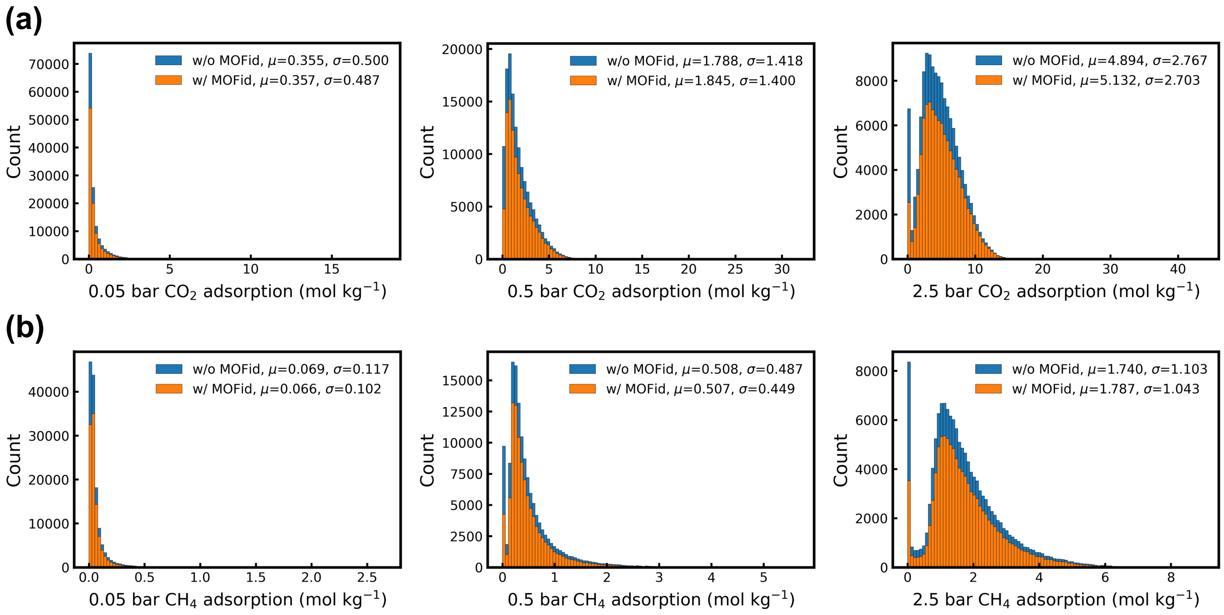

Figure S3: Histogram showing the distribution of hMOF dataset with the label of **(a)** CO_2 adsoprtion and **(b)** CH_4 adsorption at 0.05, 0.5, and 2.5 bar of pressure. Blue bars are all MOFs in the hMOF dataset. Orange bars are MOFs with MOFid available. Mean μ and standard deviation σ are reported.

4 Parameters to create SOAP feature vector

The SOAP[9] feature vector is created using the DScribe package.[10] Table S5 records the parameters for creating SOAP feature vector, which are chosen arbitrarily based on previous works.[7,11]

Table S5: Parameters used for creating SOAP feature vector

rcut	σ	nmax	lmax	average method	crossover
8	0.2	8	6	Inner average	False

The length of the SOAP feature vector varies greatly based on the number of different atom elements in the dataset. When the MOFs in a dataset includes a large variation of elements (such as QMOF, 79 elements), the SOAP feature vector will be very long and sparse. However, when the MOFs in the dataset consist of only a few different elements (such as hMOF, 11 elements), the SOAP feature vector will be relatively short and less sparse. For example, SOAP feature vector created using the 11 elements in hMOF has a length of 2772. The SOAP feature vector created using the 79 elements in QMOF has a length of 19908, given that nmax and lmax parameters are kept the same. The high accuracy of SOAP feature vector on hMOF is a direct result of the small variation of element type in the dataset. Assuming that we create the SOAP feature vectors for hMOFs using double the original number of elements (22 instead of the original 11), the length of SOAP feature vector will be doubled to 5544. The performance of XGBoost using the new SOAP feature is significantly reduced (Table S6). Therefore, we can conclude that SOAP feature is not a suitable choice when exploring the chemical space for MOFs where elements are diverse.

Table S6: Parameters used for creating SOAP feature vector

Model	# elements	SOAP vector length	hMOF 2.5 bar CH_4 adsorption
SOAP	11	2772	0.239±0.002
SOAP	22	5544	0.288±0.002
$CGCNN_{pretrain}$	-	-	0.258±0.008

5 Comparison between models' performance in QMOF dataset

Figure S4 shows the kernel density estimation (KDE) plot of the predicted band gap values of MOFs from different models. The MOFs are in a randomly selected test set from the QMOF dataset. The ground truth is the DFT calculated band gap, which is shown by the blue curve. SOAP and Stoichiometric-120 features with XGBoost model underpredict low bandgap values more frequently than CGCNN and MOFormer.

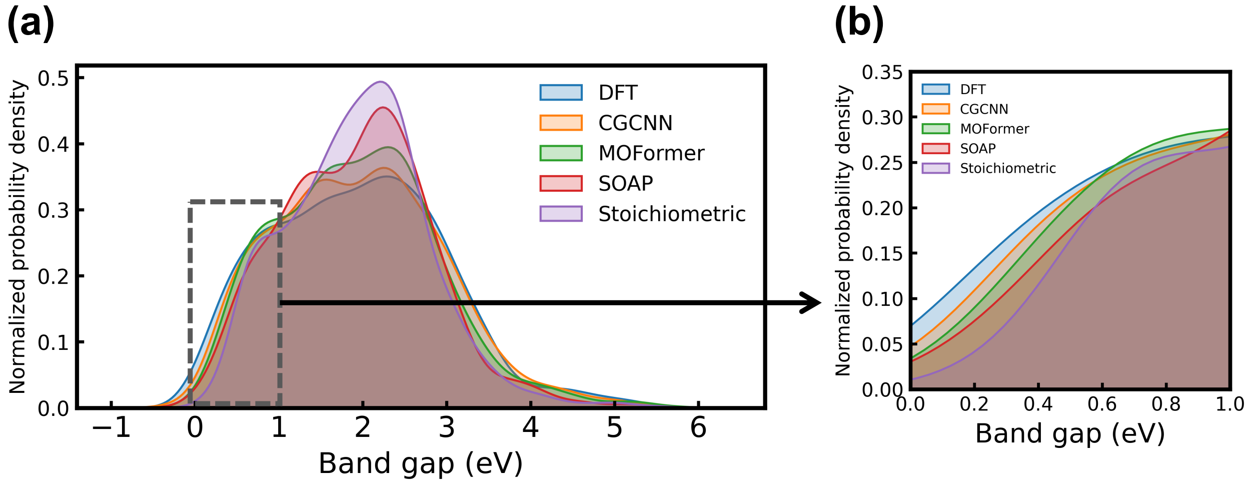

Figure S4: **(a)** The kernel density estimation (KDE) plot of the predicted band gap values of MOFs from different models. **(b)** Zoom-in view of the distribution of MOFs with band gap $\leq$ 1eV

6 Prediction accuracy with extra long MOFid or rare topology

A quantitatively evaluation of the effect of overlength MOFid on prediction accuracy is shown in Figure S5a. We plotted out the mean absolute error of MOFormer in predicting CO_2 adsorption (0.5 bar) on the test set of hMOF, with respect to the tokenized MOFid length. The test set contains 15428 MOFs. The red dotted line represents the maximum sequence length, 512, currently set in the MOFormer model. The black dashed line represents the average MAE, 0.536, over the whole test set. The average MAE of MOFs with sequence length ≤ 512 is 0.536, while the average MAE of MOFs with sequence length > 512 is 0.463. The result shown in Figure S5a does not indicate a noticeable accuracy drop with overlength MOFid.

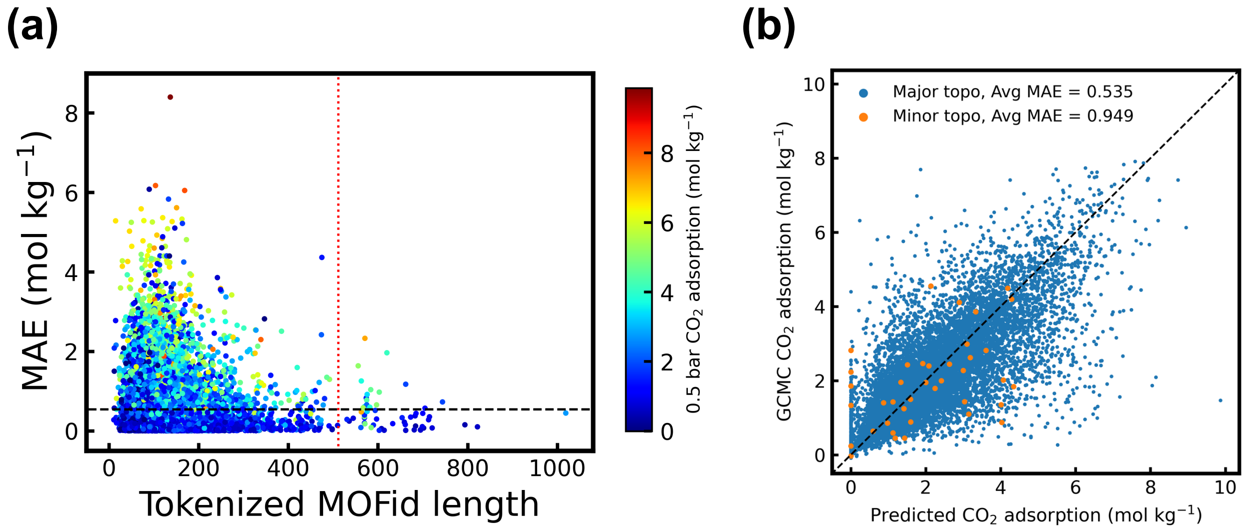

Figure S5: The performance of MOFormer on the test set of hMOF (15428 data points). **(a)** The mean absolute error of prediction with respect to the length of tokenized MOFid. Each data point is colored based on its simulated CO_2 adsorption at 0.5 bar of pressure. The red dotted line represents the maximum sequence length (512). The black dashed line represents the average MAE of the whole test set (0.536). **(b)** Predicted vs GCMC simulated CO_2 adsorption at 0.5 bar. Major topologies are the top 10 most common topologies in hMOF, which is discussed in the Figure 4 of the main manuscript. Minor topologies are the rest of the topologies in hMOF. Dashed line represents perfect prediction.

Figure S5b shows the predicted versus the GCMC simulated CO_2 adsorption (0.5 bar) of

the same test set. We define the major topologies to be the top-10 most common topologies in hMOF dataset (also shown in Figure 4 of manuscript), and the minor topologies to be the rest of topologies in hMOF. The average MAE of predicting CO_2 adsorption of MOFs with minor topologies (0.949) is higher compared with major topologies (0.535). The MOFormer model representations may fail to accurately predict the properties of MOFs with rare topologies. For structure-agnostic MOF property prediction, the problem can be alleviated by two future efforts. The first one is to increase the diversity of topologies in the training dataset if available. The second one is to design a pretraining of MOFormer model to learn the similarity/dissimilarity between topologies, which can help it infer the property of a rare topology from a common topology.

References

(1) Vaswani, A.; Shazeer, N.; Parmar, N.; Uszkoreit, J.; Jones, L.; Gomez, A. N.; Kaiser, Ł.; Polosukhin, I. Attention is all you need. Advances in neural information processing systems. 2017; pp 5998–6008.

(2) Bahdanau, D.; Cho, K.; Bengio, Y. Neural machine translation by jointly learning to align and translate. *arXiv preprint arXiv:1409.0473* **2014**,

(3) Hochreiter, S.; Schmidhuber, J. Long short-term memory. *Neural computation* **1997**, *9*, 1735–1780.

(4) Paszke, A.; Gross, S.; Massa, F.; Lerer, A.; Bradbury, J.; Chanan, G.; Killeen, T.; Lin, Z.; Gimelshein, N.; Antiga, L., et al. Pytorch: An imperative style, high-performance deep learning library. *Advances in neural information processing systems* **2019**, *32*.

(5) Xie, T.; Grossman, J. C. Crystal graph convolutional neural networks for an accurate and interpretable prediction of material properties. *Physical review letters* **2018**, *120*, 145301.

(6) Magar, R.; Wang, Y.; Barati Farimani, A. Crystal twins: self-supervised learning for crystalline material property prediction. *npj Computational Materials* **2022**, *8*, 1–8.

(7) Rosen, A. S.; Iyer, S. M.; Ray, D.; Yao, Z.; Aspuru-Guzik, A.; Gagliardi, L.; Notestein, J. M.; Snurr, R. Q. Machine learning the quantum-chemical properties of metal–organic frameworks for accelerated materials discovery. *Matter* **2021**, *4*, 1578–1597.

(8) Wilmer, C. E.; Leaf, M.; Lee, C. Y.; Farha, O. K.; Hauser, B. G.; Hupp, J. T.; Snurr, R. Q. Large-scale screening of hypothetical metal–organic frameworks. *Nature chemistry* **2012**, *4*, 83–89.

(9) Bartók, A. P.; Kondor, R.; Csányi, G. On representing chemical environments. *Physical Review B* **2013**, *87*, 184115.

(10) Himanen, L.; Jäger, M. O. J.; Morooka, E. V.; Federici Canova, F.; Ranawat, Y. S.; Gao, D. Z.; Rinke, P.; Foster, A. S. DScribe: Library of descriptors for machine learning in materials science. *Computer Physics Communications* **2020**, *247*, 106949.

(11) Fung, V.; Zhang, J.; Juarez, E.; Sumpter, B. G. Benchmarking graph neural networks for materials chemistry. *npj Computational Materials* **2021**, *7*, 1–8.
